# Protein Redox Modification as a Cellular Defense Mechanism against Tissue Ischemic Injury

**DOI:** 10.1155/2014/343154

**Published:** 2014-05-05

**Authors:** Liang-Jun Yan

**Affiliations:** Department of Pharmaceutical Sciences, UNT System College of Pharmacy, University of North Texas Health Science Center, 3500 Camp Bowie Boulevard, RES-314E, Fort Worth, TX 76107, USA

## Abstract

Protein oxidative or redox modifications induced by reactive oxygen species (ROS) or reactive nitrogen species (RNS) not only can impair protein function, but also can regulate and expand protein function under a variety of stressful conditions. Protein oxidative modifications can generally be classified into two categories: irreversible oxidation and reversible oxidation. While irreversible oxidation usually leads to protein aggregation and degradation, reversible oxidation that usually occurs on protein cysteine residues can often serve as an “on and off” switch that regulates protein function and redox signaling pathways upon stress challenges. In the context of ischemic tolerance, including preconditioning and postconditioning, increasing evidence has indicated that reversible cysteine redox modifications such as S-sulfonation, S-nitrosylation, S-glutathionylation, and disulfide bond formation can serve as a cellular defense mechanism against tissue ischemic injury. In this review, I highlight evidence of cysteine redox modifications as protective measures in ischemic injury, demonstrating that protein redox modifications can serve as a therapeutic target for attenuating tissue ischemic injury. Prospectively, more oxidatively modified proteins will need to be identified that can play protective roles in tissue ischemic injury, in particular, when the oxidative modifications of such identified proteins can be enhanced by pharmacological agents or drugs that are available or to be developed.

## 1. Introduction

Increasing evidence continues to support the concept that reactive oxygen species (ROS) and reactive nitrogen species (RNS) can exert great beneficial effects on cellular adaptation to stress challenges and cell survival [1–5]. This is particularly true in the context of ischemic tolerance that includes preconditioning and postconditioning; both of which are used to prepare tissues to tolerate injuries against lethal ischemic occurrence by triggering endogenous adaptive and defensive responses [5–13]. Evidence supporting the involvement of ROS and RNS in ischemic tolerance comes directly from the observations that administration of antioxidants before or during the induction of ischemic tolerance can abolish the protective effects of either preconditioning or postconditioning [14–20]. As one of the means that ROS/RNS work is via modifying proteins, protein redox modifications can thus execute the beneficial effects of ROS/RNS [21–26]. In this review, I will summarize evidence that protein redox modifications, in particular, reversible modifications on protein cysteine residues when induced by preconditioning or postconditioning, can serve as a cellular defense mechanism against tissue ischemic injury. Evidence presented indicates that protein redox modifications can serve as therapeutic targets in tissue ischemic injury.

## 2. Protein Redox Modifications

**Figure 1:**
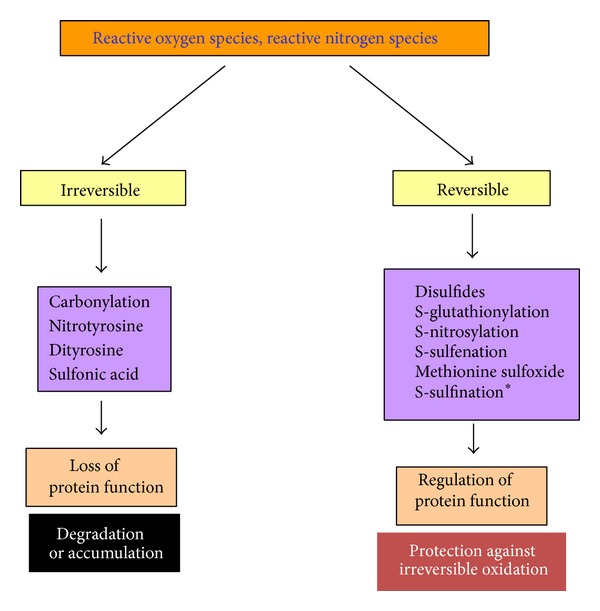
Classification of protein oxidative modifications into two categories: irreversible oxidation and reversible oxidation. *Note: only a few studies so far have reported that sulfinic acid (S-sulfinition) formation could be reversible [180, 181].

**Figure 2:**
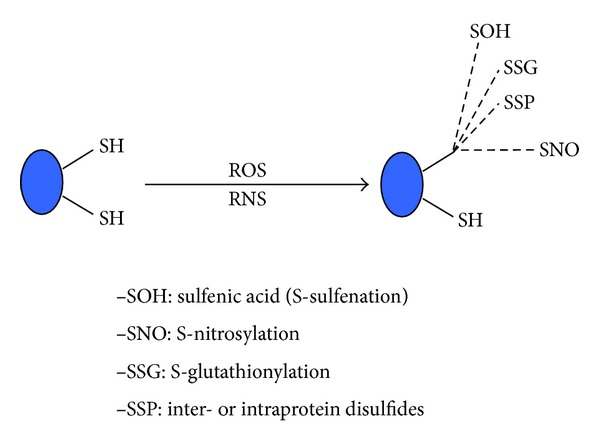
Reversible cysteine modification products that are widely studied. These products include S-sulfenation (sulfenic acid, –SOH), S-nitrosylation (–SNO), S-glutathionylation (–P–S–S–G), and either intra- or interdisulfides.

Under stress conditions, cells can produce an elevated level of reactive oxygen species (ROS) and reactive nitrogen species (RNS) [27, 28], which, in turn, can oxidize or modify proteins [29–32]. As shown in Figure 1, protein oxidation can be classified into two general categories. One is irreversible and the other is reversible. Irreversible oxidation usually leads to protein aggregation and degradation. This type of oxidation includes formation of protein carbonyls [33], nitrotyrosine [34], and sulfonic acids [35]. On the other hand, reversible protein oxidation is usually involved in redox signaling pathways and regulation of protein structure and function [36–38]. This type of oxidation often occurs on protein cysteine residues leading to formation of S-sulfenation, S-nitrosylation, disulfides, and S-glutathionylation [35, 39, 40] (Figure 2). Additionally, formation of methionine sulfoxide, involving methionine sulfoxide reductase [41–44], is also a reversible process and has been shown to be involved in protection against ischemic injury [45–47]. It should be pointed out that, strictly speaking, disulfide formation (P–S–S–P) and S-glutathionylation (P–S–S–G) are not oxidative modifications as the end products do not contain an oxygen atom like those found in S-nitrosylation (–SNO) and S-sulfenation (–SOH). Nonetheless, formation of both disulfides and glutathionylation requires the presence of reactive oxygen species such as hydrogen peroxide [48–56]. Therefore, it would be more appropriate to name these two modifications as redox modifications.

## 3. Production of Reactive Oxygen Species (ROS) and Reactive Nitrogen Species (RNS)

While all endogenous RNS originate from nitric oxide synthases, ROS can be produced by many cellular systems. Among which, mitochondria remain as a major cellular site for ROS production [28, 57–59]. It has been established that mitochondrial complexes I and III are the major two sites for mitochondrial ROS production [57, 58]. Other enzyme systems in mitochondria that can generate ROS include complex II [60], *α*-keto acid dehydrogenase complexes that contain dihydrolipoamide dehydrogenase [61–65]. Outside mitochondria, NADPH oxidase [66, 67], xanthine oxidase [68, 69], and cytochrome P-450 enzymes [70] can also generate ROS. It has been recently reported that the *α*-keto acid dehydrogenase complexes can generate more ROS than complex I [71]. The initial species is always a superoxide anion, which can be dismutated to H_2_O_2_ [72–74]. The latter can decay to form hydroxyl radical in the presence of metal ions [75]. Additionally, when superoxide meets nitric oxide, peroxynitrite is formed [76, 77]. Both hydroxyl radical and peroxynitrite are known to be highly reactive towards proteins [78, 79].

## 4. Balance and Imbalance between Oxidants and Antioxidants

Production of ROS and RNS is a well-controlled process under normal conditions [80, 81]. This is because cells have a variety of antioxidant defense systems. These include but are not limited to superoxide dismutase, catalase, glutathione peroxidase, thioredoxin, and peroxiredoxin [27]. Moreover, there are also small antioxidant molecules such as vitamin C, vitamin E, glutathione, and coenzyme Q [27]. Under normal physiological conditions, a balance between ROS production and antioxidant defense is well maintained [53, 82]. However, under stress or pathophysiological conditions, more ROS and RNS can be produced that can overwhelm the cellular antioxidant defense system, leading to severe oxidative stress and oxidative damage [80, 83]. On the other hand, intentionally induced oxidative stress can serve as a defense mechanism against further oxidative challenges [80, 83–87]. This is known as hormetic effect [88–90] or “positive oxidative stress” and are often explored as a protective approach in ischemic tissue injuries [26], a phenomenon often termed as ischemic tolerance that includes both preconditioning and postconditioning [7, 17, 91].

## 5. Ischemic Tolerance: Preconditioning and Postconditioning

Preconditioning is a prophylactic approach, which often involves noninjurious stimulation of the tissues that are of interest [7, 17, 91]. Such stimulation can prepare the tissues to resist further challenges that are lethal [7, 17, 91]. Induction of preconditioning can be achieved by many ways, including short episodes of ischemia reperfusion [92], treatment with chemicals or drugs that are often inhibitors of mitochondrial electron transporter complexes [26, 93], hyperoxia [94, 95], and hypoxia [96], as well as remote preconditioning [97]. Remote preconditioning means that the tissues that receive preconditioning can defend other tissues against ischemic injuries. Therefore, the target to be preconditioned and the target to be protected are not the same in the settings of remote preconditioning. As opposed to that of preconditioning, postconditioning is the interruption or intervention at the onset of reperfusion after an ischemia has occurred [98–102]. Therefore, postconditioning may be more clinically relevant as ischemic occurrence is generally not a predictable event. Nonetheless, preconditioning is still intensively studied because investigating how tissues respond to preconditioning may identify endogenous therapeutic targets for treatment of ischemic injury [103, 104]. Moreover, both preconditioning and postconditioning have been shown to involve similar signaling pathways or trigger similar defense mechanisms [100, 105–107].

## 6. Reversible Protein Cysteine Modifications and Ischemic Tolerance

In the context of ischemic tolerance including preconditioning and postconditioning, cysteine redox modifications have been explored extensively. This is because cysteine oxidation is closely associated with cellular redox potential reflected by the ratio between GSH/GSSG and NADH/NAD^+^ [108, 109]. Moreover, cysteine residues can undergo reversible modifications that are involved in an “on and off” switch during stress conditions [36, 110–112]. Therefore, reversible cysteine modifications are often involved in regulating redox signaling pathways and protein function [37, 113, 114]. Accordingly, I will cover only reversible cysteine modifications and their protective roles in ischemic injury in in this review. These include S-sulfenation, S-nitrosylation, S-glutathionylation, and disulfide formation. But, before discussing each of the four modifications, I would like to briefly introduce a general method for analysis of reversible cysteine modifications as the method has contributed significantly in the paradigms to be presented below in this review.

## 7. General Detection Method for Reversible Cysteine Modifications

**Figure 3:**
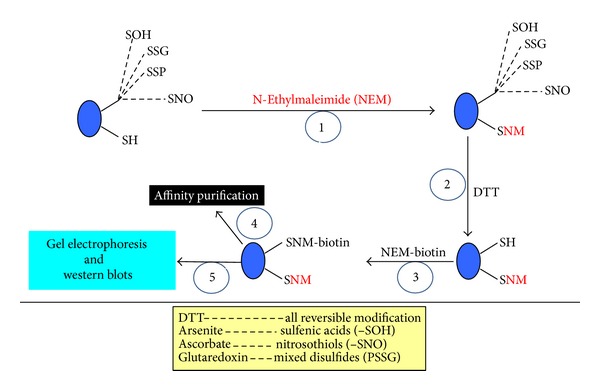
Analysis of reversible cysteine redox modifications. What is shown is a one of the popular methods generally called a “biotin switch” assay, which involves alkylating the free thiol groups (step 1), reducing the modified cysteine residues using specific reductant for each oxidation product (step 2), and relabeling of the newly generated free thiol groups using biotin conjugated probes (step 3). Following biotinylation, the samples can be further analyzed by either western blot (step 5) or affinity purification (step 4). Note that protein sulfenic acids can be directly labeled by dimedone conjugated biotin probes as described in the text.

As cysteine oxidation does not involve a change in optical density of the modified proteins, a probe is always needed for the detection of cysteine modifications [115]. In fact the approaches are quite similar for S-sulfenation, S-nitrosylation, and S-glutathionylation.  Figure 3 shows one of the general procedures for detection of cysteine oxidation products. This widely used method is often called biotin switch assay [116]. The steps involve blocking unmodified cysteine residues with alkylating reagents such as N-ethylmaleimide (NEM), reducing the modified cysteine residues using a specific reducing reagent for each modified species [38, 117]. For example, ascorbic acid is used for the reduction of S-nitrosylation [118], arsenite is used for the reduction of S-sulfenation [119], and glutaredoxin is used for the reduction of S-glutathionylation [120, 121]. This is followed by relabeling of the reduced cysteine residues using biotin conjugated with an alkylating reagent such as NEM. This approach not only facilitates gel-based detection as biotin can be readily recognized by streptavidin, but also can be conducive to affinity purification of the modified proteins. Additionally, NEM-biotin labeling can also pinpoint the site of modifications when used in conjunction with mass spectrometric peptide sequencing. It should be noted that, for the detection of protein sulfenic acids, biotin conjugated dimedone probes have been developed that only reacts with –SOH [122, 123]; therefore no blocking and reducing steps are needed. For the detection and quantification of S-glutathionylation, the enzyme glutaredoxin is needed in the presence of GSH. DTT and 2-mercaptoethanol are nonspecific reducing reagents; hence they are not good for a specific modifying species.

## 8. Paradigms of Reversible Protein Cysteine Modifications as a Defense Mechanism in Ischemic Injury

### 8.1. S-Sulfenation (–SOH)

S-Sulfenation or protein sulfenic acid (–SOH) is now attracting increasing attention because this cysteine redox modification product can now be readily trapped and quantified [115, 123]. Moreover, although once considered a transient product of cysteine oxidation adduct, stable –SOH has been found to exist that plays an “on and off” switch in regulating protein function and redox signaling [40]. An elegant model of protein sulfenic acid formation in protecting ischemic tissue injury is the enzyme aldose reductase that has been studied thoroughly by Dr. Bhathagar's group at University of Louisville. This group initially found that AR could be activated by ischemic reperfusion in the heart, and this activation was due to the formation of a sulfenic acid on cysteine residue 298 [124]. Furthermore, this sulfenation process of cysteine 298 was found to be achieved by peroxynitrite [125], a highly reactive species formed between superoxide anion and nitric oxide [126]. The group next found that this activation of AR via cysteine sulfenic acid formation was regulated by the PI3K/AKT/eNOS signaling pathway [125]. As this pathway is known to be involved in protection against ischemic injury [127, 128], AR activation by sulfenic acid formation on cysteine 298 thus is suggested to be involved in cardioprotection against cardiac injury, which is further supported by the observation that AR inhibitors such as sorbitol or tolrestat, when applied before ischemia or at the onset of reperfusion, hindered postischemic recovery in the heart [125]. Interestingly, as this seems to be the end of the story, this laboratory went further and demonstrated that formation of AR–SOH on cysteine 298 during cardiac ischemia reperfusion could be reversed back to AR–SH [129], which involved two enzymes, glutathione S-transferase converting AR–SOH to AR–SSG, and glutaredoxin converting AR–SSG to AR–SH [129]. Therefore, both enzymes may be involved in regulation of AR–SOH reduction when tissue oxygen and nutrient supply is resumed after an ischemic incident.

### 8.2. S-Nitrosylation (–SNO)

Protein cysteine nitrosylation (P-SNO), another form of reversible modification, has been studied by numerous investigators. The role of this modification has been thought to be equivalent to that of protein phosphorylation [130, 131]. It not only has detrimental effects on protein function and cell survival [132, 133], but also can exert beneficial effects under a variety of pathophysiological conditions [134, 135]. In the context of tissue ischemic injury, it has been found that overall protein –SNO, in connection with the activation of the PI3K/AKT signaling pathway, increases after postconditioning in the heart [136], indicating that nitrosylation of individual proteins play a protective role in ischemic injury. This is indeed the case as presented in the following two examples.

#### 8.2.1. S-Nitrosylation of TRIM72 at Cysteine-144 Is Cardioprotective

Tripartite motif-containing protein 72 (TRIM72) is a membrane repair protein that can undergo posttranslational modifications leading to its either activation or degradation. Using the biotin switch assay shown in Figure 3, Kohr et al. reported that TRIM72 exhibited an elevated level of SNO at cysteine-144 upon ischemic preconditioning [137]. As ischemic preconditioning is an established approach for cardioprotection against ischemic injury [138], the authors hypothesized that increase in TRIM72's cysteine-144 nitrosylation protects against cardiac ischemic injury. The authors tested the hypothesis by mutating C144 to a serine residue (C144S) in a tissue culture system using HEK-293 cells that lack TRIM72. This mutation would abolish the proteins S-nitrosylation at C144, hence changing the protein's property and function. Indeed, the authors found that after the mutation, protein levels of TRIM72 (wildtype) but not TRIM72-C114S (mutant) were decreased upon H_2_O_2_ treatment, and this decrease correlated with enhanced H_2_O_2_-induced cell death in the wild type cells. Moreover, treatments of the cells with an S-nitrosylating agent S-nitrosoglutathione (GSNO) [139] could maintain TRIM72's protein level and reduce cell death. The authors further demonstrated that GSNO induced TRIM72 nitrosylation stopped ischemia reperfusion triggered decrease in TRIM72 levels and decreased infarct size in heart ischemia reperfusion. Thus, cys144-SNO of TRIM72 prevents degradation of TRIM72 upon ischemic challenge and thus preserves its membrane repair capacity.

#### 8.2.2. S-Nitrosylation of Mitochondrial Complex I ND3 Subunit Participates in Cardioprotection against Ischemic Injury

Complex I is the electron entry point in the mitochondrial electron transport chain. It has at least 45 subunits in the mammalian systems and many of them are redox sensitive [140–142]. Dysfunction of complex I is thought to be a causal factor in the pathogenesis of many mitochondrial diseases including ischemic injury [143–145]. Recently, Chouchani et al. reported that S-nitrosylation of the complex I subunit ND3 is involved in cardioprotection against ischemic insult [146]. The authors reported that S-nitrosylation of ND3-cysteine-39 inhibited complex I activity and slowed mitochondrial recovery at the initial minutes of reperfusion, hence attenuating ROS generation upon sudden oxygen resupply, leading to less oxidative damage and tissue necrosis. Interestingly, ND3 only became accessible to nitrosylation after an ischemic insult as mitoSNO, a membrane permeable nitrosylating agent, could only provide the protective effect at the onset of reperfusion via ND3 cysteine-39 nitrosylation. As mitoSNO was applied during reperfusion and its protective effect could only be observed when administered at the onset of reperfusion, this study provides an elegant postconditioning paradigm whereby S-nitrosylation could serve as one mechanism contributing to postconditioning-induced ischemic tolerance.

### 8.3. S-Glutathionylation

Well-defined roles of protein S-glutathionylation in ischemic tolerance have not been clearly reported in the literature. Nonetheless, there are direct link that protein S-glutathionylation induced by preconditioning prevents cell death and enhances cell survival. The results of two studies will be summarized here. The first one is S-glutathionylation of mitochondrial adenine nucleotide translocase (ANT) induced by carbon monoxide preconditioning [147]; and the second one is S-glutathionylation of ryanodine receptor 2 induced by tachycardia preconditioning via elevation of NADPH oxidase activity. In the first study, Queiroga et al. reported that carbon monoxide prevents mitochondrial permeability transition pore opening and cell death via S-glutathionylation of ANT [147]. In particular, using nonsynaptic mitochondria isolated from rat brain and primary astrocytes prepared from the cortex of neonatal rats, the authors found that carbon monoxide could partially inhibit loss of mitochondrial membrane potential, the opening of mitochondrial membrane permeability transition pore, mitochondrial swelling, and cytochrome c release. To understand the underlying mechanisms, the authors further found that carbon monoxide could modulate ANT activity as ADP/ATP exchange rate was enhanced. As ANT is part of the mitochondrial membrane permeability transition pore [148], this enhancement of ANT activity thus also prevented pore opening. Moreover, it was further found that the modulation of ANT activity was due to ANT glutathionylation caused by carbon monoxide-induced ROS production. It should be noted that the site of glutathionylation on the ANT molecule was not identified in this study.

In the second study, Sánchez et al. reported that while electrically induced tachycardia can effectively create myocardial preconditioning, the mechanisms remain elusive [149]. Therefore, the authors set out to elucidate the underlying mechanisms. Focusing on sarcoplasmic reticulum (SR) isolated from dog cardiac ventricular muscle, they found that preconditioning tachycardia increased NADPH oxidase activity by nearly 200% as measured by NADPH dependent superoxide production. This increase in enzymatic activity was due to the enhanced association of rac1 with the NADPH oxidase cytosolic subunit p47 (phox) to the microsomal fraction without altering the content of the enzyme's membrane subunit gp91 (phox). As an elevated level of superoxide can induce protein S-glutathionylation, the author further found that cardiac ryanodine receptor 2 (RyR2) was S-glutathionylated under their experimental conditions. Conversely, when catalase, superoxide dismutase, and NADPH oxidase inhibitors were added in the experimental system, RyR2 S-glutathionylation was greatly attenuated, indicating a potential link between RyR2 glutathionylation and tachycardia preconditioning. Interestingly, this same laboratory further reported that exercise could also produce a preconditioning effect by increasing NADPH oxidase activity and RyR2 S-glutathionylation [150]. Similar to the ANT studies presented above, the site of modification on Ry2R was also not pinpointed in this study.

### 8.4. Disulfides

Numerous studies have demonstrated that oxidative stress-induced disulfide formation can be beneficial to cell survival [151–155]. An excellent study by Fourquet et al. [156] presented a well-delineated role of protein disulfide formation in activation of the Nrf2 signaling pathway that regulates the expression of the second phase defensive enzymes such as hemeoxygenase-1 (HO-1) and NAD(P)H dehydrogenase quinone-1 (NQO-1) [157]. Using Hela cells treated with H_2_O_2_, nitric oxide, and hypochlorite, the authors found that Keap1, a protein that controls the fate of Nrf2, can form intramolecular disulfides, leading to release and nuclear translocation of Nrf2. The authors further found that cysteine-151 of Keap1 was involved in disulfide bond formation between two molecules of Keap1, forming a Keap1 homodimer. This formation of Keap1 homodimer is important for Nrf2 release from the Keap1-Nrf2 complex as mutation of cysteine-151 led to an unstabilized form of Nrf2. Additionally, the authors also found that, when the thioredoxin and glutathione pathways were inactivated, Keap1 intramolecular disulfide bond formation was constitutive, leading to a stable Nrf2 molecule in the cell. Therefore, this study further demonstrates that Keap1 cysteine-151 disulfide bond formation is at least one of the mechanism by which cells utilize to resist ischemic injury by upregulating the second phase antioxidative proteins [158–166], which include thioredoxin reductase, glutamate-cysteine ligase (GCL), glutathione S-transferase, HO-1, and NQO-1, [157, 167–171].

## 9. Summary and Perspective

Protein redox modification is a double-edged sword. While there is no doubt that protein redox modifications can have detrimental effects on cell survival [172–179], there is also increasing evidence, as summarized in this review, that redox modification of certain proteins, when induced purposely by approaches that trigger positive oxidative stress [26], can play a protective role in tissue ischemic injury. Studying how proteins respond to oxidative modifications in the settings of preconditioning and postconditioning, may identify novel proteins as potential therapeutic targets for treatment of ischemia-related diseases, in particular, when such modifications can be enhanced by pharmacological agents.
